# Reproductive Health Among School Employees in Vijayapura, Karnataka

**DOI:** 10.7759/cureus.75535

**Published:** 2024-12-11

**Authors:** Rajasri G Yaliwal, Shreedevi Kori, Aravind V Patil, Shailaja R Bidri, Gauri Bankapur, Swati A Talwade

**Affiliations:** 1 Obstetrics and Gynecology, Shri B M Patil Medical College Hospital and Research Centre, BLDE (Deemed to be University), Vijayapura, IND; 2 General Surgery, Shri B M Patil Medical College Hospital and Research Centre, BLDE (Deemed to be University), Vijayapura, IND

**Keywords:** contraception literacy, pap smear test, screening program, uterine cervical cancer, visual inspection with acetic acid (via)

## Abstract

Background

Cervical cancer typically progresses over 10-20 years, making it a preventable disease and underscoring the importance of screening. In low-resource settings, Papanicolaou (Pap) smears and visual inspection with acetic acid (VIA) serve as primary screening tools. This study was conducted as part of the noncommunicable disease camps organized by the government of Karnataka, India. Additionally, it aimed to evaluate the knowledge, attitudes, and practices related to contraception among women of reproductive age.

Methods

This cross-sectional study was conducted from June to August 2022 in primary health centers, community health centers, and government schools across all the Talukas of Vijayapura District, Karnataka, India. A total of 6,257 women participated in the study. The researchers interviewed all participants, obtained detailed medical histories, and performed pelvic examinations as part of the evaluation. Additionally, they conducted both VIA and Pap smears.

Results

A total of 6,257 women were sensitized for cervical cancer screening, of whom 5,114 registered, resulting in an acceptance rate of 81.73%. However, 1,143 participants (18.27%) refused screening, with the most common reason being unwillingness to undergo an examination. Only 3,316 women (53.91%) were aware of cervical cancer, and just 1,689 (27.1%) understood that it could be detected at an early stage. The VIA results indicated that 9.1% (n = 865) of the women tested positive. Among those screened, seven women (0.1%) were found to have a high-grade squamous intraepithelial lesion. Of these, four underwent colposcopy-guided biopsy, while three were lost to follow-up; all four biopsies were negative for malignancy. Additionally, two women presented with apparent cervical growths. Regarding contraceptive practices, 2,690 women (43.3%) expressed support for using contraception, with the permanent method of sterilization being the most preferred by 1,990 women (74.13%).

Conclusions

Screening with basic investigations such as Pap smears and VIA plays a crucial role in identifying premalignant and malignant lesions at their earliest stages, enabling timely treatment to reduce morbidity and mortality. Additionally, most women opt for permanent contraception once they have completed their families.

## Introduction

Cervical cancer, with an incidence rate of 16%, is the second most common malignancy among Indian women and ranks among the most prevalent gynecological cancers globally. It has a mortality rate of 7.2 deaths per 10,000 woman-years and an incidence rate of 13 cases per 10,000 woman-years [1]. According to GLOBOCAN 2020, it is the second leading cause of death among women due to malignancies [1]. The relative five-year survival rate is approximately 46% (IQR: 34-60%) [2], primarily because about 80% of cases are diagnosed at an advanced stage, contributing to significant mortality. Globally, approximately 80% of cervical cancer cases occur in developing and low-income countries [3].

Social and cultural barriers, coupled with a lack of knowledge and information about cervical cancer screening, play a critical role in the high disease burden. Since the natural progression from mild dysplasia to cervical cancer generally takes 10-20 years, timely screening is essential for early detection and prevention [4]. The Papanicolaou (Pap) smear test is pivotal in identifying precancerous changes in the cervix before malignancy develops, with a sensitivity of 50-75% and a specificity of 98-99% [5]. Another key screening tool in low-resource settings is visual inspection with acetic acid (VIA), which is effective for identifying early-stage cervical cancer [6].

The 2016 National Programme for Prevention and Control of Diabetes, Cardiovascular Diseases, and Stroke (NPCDCS) guidelines in India introduced population-based cervical cancer screening using VIA, recommending that women aged 30-65 years be screened every five years [7]. The Federation of Obstetric & Gynaecological Societies of India (FOGSI) also recommends VIA as the preferred screening method in low-resource settings.

In November 2020, the WHO launched the Global Cervical Cancer Elimination Initiative, with the goal of reducing cervical cancer incidence to less than four cases per 100,000 woman-years in every country. The initiative’s “90-70-90” target aims for 90% of adolescent girls to be vaccinated by age 15, 70% of women aged 45 years to undergo at least two high-performance screening tests, and 90% of women diagnosed with cervical precancerous lesions or cancer to receive treatment by 2030 [8]. As part of this goal, the WHO has emphasized the need for ongoing enhanced surveillance and monitoring of cervical cancer.

Hesitation to seek medical attention in low-resource settings is multifactorial, involving lack of privacy, social and cultural barriers, high healthcare costs, policy and healthcare system issues, lower social status, and limited access to healthcare [9]. Sexually transmitted infections (STIs) and reproductive tract infections are common causes of reproductive health issues. Over 340 million new STIs occur annually worldwide, with 11 million of those cases in South and Southeast Asia. Young women are at higher risk due to lower immunity and greater cervical ectopy. A study on gynecological and sexual disorders among rural women in India found that 55% of women reported gynecological problems, and 92% had one or more STIs [10].

India, with a population of 1.42 billion and a fertility rate of 2.139, faces significant challenges related to overpopulation. The prevalence of contraception is an indicator of women's empowerment and access to reproductive health care [11]. While Indian women are increasingly aware of family planning options, overpopulation remains a key concern. One of the primary objectives of this study was to evaluate the knowledge, attitudes, and contraceptive practices among women of reproductive age. Despite national screening efforts, limited data exist on reproductive health and cancer screening practices among school employees in this region.

## Materials and methods

The study was conducted in the district of Vijayapura, Karnataka, India, as part of the noncommunicable disease camps organized by the government of Karnataka in collaboration with Shri B M Patil Medical College Hospital and Research Centre, BLDE (Deemed to be University), Vijayapura, India. The study was registered in the Clinical Trial Registry of India under CTRI number CTRI/2022/07/044308. Institutional Ethical Clearance was granted on May 27, 2022, with IEC number BLDE(DU)/IEC/628/2022-23. A total of 31 camps were conducted across primary health care centers, community health care centers, and government schools in all the Talukas of Vijayapura District.

The inclusion criteria consisted of all women aged 18 years or older who were currently or had been married. Women who were unmarried, those known to have been treated for cervical cancer, and pregnant women were excluded from the study.

Data collection 

Healthcare workers, doctors, postgraduates, and interns were trained to take a thorough gynecological history from the women attending the camp. Staff were also trained in performing Pap smears and VIA. Junior residents practiced VIA and Pap smears in the OPD before conducting them in the field. An adequate number of Cusco’s speculums, Ayre’s spatulas, endocervical cytobrushes, and cotton-tipped swabs for VIA were procured for the camp. Fresh 5% acetic acid was prepared on-site, and 2% glutaraldehyde was used for sterilization.

Written informed consent was obtained from all participants. Urine samples, antibiotics, and environmental monitors were excluded from the study on the day of the camp. A Cusco’s bivalve vaginal speculum was inserted through the cervix, and the patient was positioned in the lithotomy position. The cervix was examined for any apparent lesions, followed by a Pap smear examination. The specimens were sent to the cytology laboratory of the institute’s Department of Pathology and stained using the Pap procedure.

For VIA, 5% acetic acid was applied to the cervix, and any aceto-white spots were observed after one minute. Other identifiable lesions were also recorded. Written consent was obtained from the patients, and the data was recorded in a prevalidated digital format.

Data analysis 

Each attribute will be accompanied by a descriptive summary, including the relevant numbers and percentages for categorical data.

## Results

The team conducted 31 screening camps in the district of Vijayapura, where they sensitized 6,257 women for cancer screening. A total of 5,114 women registered for screening, resulting in an acceptance rate of 81.73%. Most participants were educated, with 5,568 women (89.1%) having received formal education. In terms of gravidity, 10.2% were primigravida (n = 524), 39% were gravida 2 (n = 1,994), 25.3% were gravida 3 (n = 1,293), and 12.6% were above gravida 3 (n = 645). Additionally, 14.3% of the total population (n = 894) had a post-hysterectomy status.

Sociodemographic details of the women attending the camp are provided in Table 1.

**Table 1:** Sociodemographic characteristics of women in NCD camp NCD, noncommunicable disease

Attribute	Frequency	Percentage
Education		
Illiterate	558	10.9
Primary	1,156	22.6
Secondary	1,314	25.7
Graduate and above	1,393	27.2
Parity		
0	282	5.5
1	524	10.2
2	1,994	39
3	1,293	25.3
>3	645	12.6

The most common complaint observed in the majority of cases was white discharge, accounting for 22.1% (n = 1,130), followed by abnormal uterine bleeding at 14.5% (n = 743), as shown in Table 2.

**Table 2:** Most common complaints

Complaint	Frequency	Percentage
Abnormal uterine bleeding	743	14.5
White discharge	1,130	22.1

Of the participants, 2,690 (43.3%) favored contraception, with 1,990 (74.13%) preferring permanent sterilization. Various methods of contraception are detailed in Table 3.

**Table 3:** Contraception use DMPA, depot medroxyprogesterone acetate; IUCD, intrauterine contraceptive device; OCP, oral contraceptive pill

Type of contraception	Frequency	Percentage
None	2,899	56.7
DMPA	1	0.019
IUCD	149	2.91
Laparoscopic ligation	197	3.85
OCP	226	4.41
Tubectomy	1,642	32.1

Only 3,372 (53.91%) participants had heard of cervical cancer, and of those, 913 (27.1%) knew that it could be detected in the early stages. However, 583 (17.3%) women refused screening, with the most common reason being unwillingness to undergo the examination. Nearly 2,878 (46%) participants had never heard of cervical cancer.

Table 4 discusses the various other lesions observed during the examination, with hypertrophy of the cervix (31.46%) and ectropion of the cervix (32.96%) being the most prominent.

**Table 4:** Other lesions

Type of lesion	Frequency
Polyp	1,275
Ectropion	1,686
Hypertrophy	1,609
Excessive white discharge	1,506
Prolapse	1,240
Unhealthy cervix	1,285
Growth on cervix	33

Seven women screened were found to have high-grade squamous intraepithelial lesions (0.1%). These women were contacted and referred to Shri B M Patil Medical College Hospital and Research Centre, BLDE (Deemed to be University), Vijayapura, India. Four of the women underwent colposcopy-guided biopsy, while three were lost to follow-up. All four biopsies were negative for malignancy. Two women exhibited obvious cervical growth and were referred to BLDE for further management. The VIA positive percentage was 9.1% (n = 463) (Table 5).

**Table 5:** VIA results VIA, visual inspection with acetic acid

VIA results	Frequency	Percentage
Positive	463	9.1
Negative	4,585	89.6
Suspicious	66	1.3

## Discussion

This study is a classic example of a large-scale community-based screening program using the camp approach. The results will assist the Indian government in prioritizing high-disease prevalence zones under the NPCDCS 2017, a population-based cervical cancer screening program [12].

The findings are consistent with a hospital-based study by Hegde et al., conducted in Karnataka, which showed a high rate of VIA and Pap smear positivity (12% and 11.7%, respectively). In our study, the VIA positivity rate was 9.1%, and the Pap smear positivity rate was 9.2%, which aligns with these findings [13]. Our study also revealed that women exhibiting symptoms of STIs during rectal examination were more likely to have precancerous cervical lesions compared to asymptomatic women who tested positive for VIA. These results are consistent with studies in Tanzania, Uganda, northern Ethiopia, and southwestern Ethiopia, which demonstrated a significant association between STDs and premalignant cervical lesions in women [14]. The link between unprotected sex and precancerous cervical lesions underscores this relationship.

In line with the research of de Oliveira et al., who found that socioeconomic factors play a central role in sustaining sterilization as the dominant method in Indian family planning, our study also found that sterilization (74.13%) was more common than other contraceptive methods (Table 3). Therefore, overcoming socioeconomic barriers and implementing cost-effective strategies - such as utilizing the media to promote short-term contraceptive options - should be key objectives of reproductive health programs [15].

Nearly 46% (2,878) of participants in our study had never heard of cervical cancer, highlighting the continued gaps in awareness despite national efforts, including mass media campaigns, community-based cervical screening, and health care worker training. This finding is consistent with the work of Misra et al., who noted poor cervical cancer awareness in rural populations [16]. However, our study also showed that 81.73% of participants had heard of cervical cancer, a higher rate likely due to the thorough counseling and the high educational status of many attendees. A study by Tripathi et al. in rural India found poor knowledge, attitudes, and practices regarding cervical cancer awareness, its signs and symptoms, detection methods, and available treatments. The main contributing factors were sociocultural, economic, and environmental, closely linked to age, education level, and family per capita income [17].

Limitations

As participation was voluntary, selection bias may have been introduced. Additionally, convincing women to undergo pelvic examinations was challenging due to cultural and social beliefs, leading to reluctance in some cases. As a result, a significant number of participants were lost to follow-up, which may have impacted the overall results.

## Conclusions

Screening with basic investigations, such as Pap smears and VIA, is crucial for identifying premalignant and malignant lesions at an early stage. Early detection and treatment significantly reduce morbidity and mortality. Most women in the study preferred permanent contraception after completing their families. There is a clear need for targeted awareness campaigns to enhance knowledge about cervical cancer screening and reproductive health practices, particularly among school employees.
